# On the Cure of Vesico-Vaginal Fistula

**Published:** 1847-05

**Authors:** Joseph Pancoast

**Affiliations:** Professor of Anatomy in Jefferson Medical College; Philadelphia


*On the Cure of Vesico-Vaginal Fistula.* By JOSEPH PANCOAST, M. D., Professor of Anatomy in Jefferson Medical College.

To Professor Huston.

*Dear Sir*,—At your request I send you a brief notice of a new mode of operation for the cure of urethro and vesico-vaginal fistula, which I have successfully employed in two cases, a more detailed account of which I propose hereafter to present you with. The two operations above alluded to, were respectively on patients of Professor Meigs, and Dr. Condie. In one case, there was a complete destruction of a cross section of the whole urethral structure, at its juncture with the neck of the bladder; in the other there was an elongated orifice in the *bas fond* of the bladder, which would more than admit the end of the finger.

"The peculiarity of the operation, consists virtually in attaching the two sides of the anormal opening firmly together, on the principle of the tongue and groove, so as to get four raw surfaces in contact, and thus increase the probabilities of union by first intention. For this purpose it is necessary that the margins of the fistula should have considerable thickness; and when not found in this state, they are to be thickened by repeated applications of lunar caustic, or, better still, of the actual cautery.

"Having exposed the fistulous orifice as thoroughly as possible with a Charriére's speculum from which the sliding blade has been removed, an assistant at the same time drawing the vestibulum well up towards the front of the pubis, my first object in the operation is to split the most posterior margin of the fistula to the depth of half an inch, with a sharp-pointed sabre-shaped bistoury. I next pare off the edges of the other lip of the fistula, so as to bring it into a wedge-shape; first reverting it as much as possible with a small blunt hook, and trimming off the mucous membrane on the side next the bladder with the curved scissors or scalpel, and then detaching in like manner, the vaginal mucous membrane, to the breadth of three-quarters of an inch, along the whole extent of the lip. This is a very difficult but most important part of the process; and great care should be taken to obtain a sufficient extent of raw surface, at the two angles of the fissure, where the lips will rest merely in apposition. Having checked the bleeding by the use of astringent applications, my next object is to insert the raw wedge or tongue into which one of the lips of the fistula has been converted, into the groove which has been cut in the other, and hold them in close connection. This I accomplish, by the means of a peculiar suture that might be called the plastic, and in the same way that I have described its application in reference to some plastic operations, in my Operative Surgery, and in the American Journal of the Medical Sciences, for October, 1842. The suture threads are to be passed with short, sharp, curved needles, held in Physick's artery forceps with the handles made of twice the ordinary length.

"When the sutures are knotted firmly, the tongue or wedge will be found immovably imbedded in the groove. The sutures I leave for two weeks or more, or until they become loose. A gum catheter should be kept in the bladder to prevent the accumulation of urine. To keep the inflammation from running to a destructive height, a bladder of cold water should be applied for thirty-six hours to the vulva.

"On the second day I direct the frequent injection of a solution of Zinci. Sulph. into the vagina, in order to increase the tone of the parts. On the third or fourth day I apply to the line of union a solution of lunar caustic with a camel's hair pencil. This application should be made twice in the twenty-four hours, the solution being gradually increased in its strength. Union by first intention may be expected to take place under this treatment to a considerable extent; at such points as it should fail to occur, union by second intention is to be promoted by the use of lunar caustic in substance, so as to raise a bed of granulation on the raw surfaces of the lips, while they are held in contact by the plastic suture. Occasionally, where the fissure is large, it will become necessary to repeat the process after a partial success has been obtained by the first operation.

*Philadelphia, April* 8, 1847.

